# Diagnostic Accuracy of a Novel Chemiluminescent Immunoassay for *Strongyloides stercoralis* in a Non-Endemic Clinical Setting

**DOI:** 10.3390/diagnostics16193227

**Published:** 2026-10-06

**Authors:** Sara Roose, Lorenza Sanfilippo, Stefano Tais, Sara Negrelli, Eleonora Rizzi, Giulia Gambini, Francesca Tamarozzi, Francesca Perandin, Dora Buonfrate

**Affiliations:** 1 Department of Infectious Tropical Diseases and Microbiology, IRCCS Sacro Cuore Don Calabria Hospital, 37024 Negrar di Valpolicella, Verona, Italy; 2 Clinical Research Unit, IRCCS Sacro Cuore Don Calabria Hospital, 37024 Negrar di Valpolicella, Verona, Italy

**Keywords:** *Strongyloides stercoralis*, serology, chemiluminescent immunoassay, diagnostic accuracy, diagnosis, Bayesian latent class analysis

## Abstract

**Background/Objectives:** *Strongyloides stercoralis* is a soil-transmitted helminth that can cause severe disease in immunocompromised individuals. In non-endemic clinical settings, recommended diagnostic strategies rely on serology alone or in combination with a sensitive fecal test, maximizing sensitivity to avoid missed infections. The *S. stercoralis* VirClia^®^ IgG monotest (Vircell, Spain) is a novel automated chemiluminescent immunoassay (CLIA) that detects IgG antibodies against the recombinant NIE and SsIR antigens. This study evaluated its diagnostic accuracy (sensitivity and specificity) in a non-endemic clinical setting. **Methods:** In this monocentric retrospective study, 245 serum samples from the Tropica Biobank of a referral centre for parasitic diseases in Italy were analyzed. Samples originated from individuals from Africa (n = 85), America (n = 31), Asia (n = 53), and Europe (n = 76). Diagnostic accuracy was estimated using Bayesian latent class analysis based on a model including five routinely used diagnostic tests. **Results:** The *S. stercoralis* VirClia^®^ IgG monotest showed a sensitivity of 95.0% (95% credible interval [CrI]: 90.1–98.2%) and a specificity of 90.8% (95% CrI: 85.1–96.0%) for the tested population. **Conclusions:** In immunocompetent individuals with a risk of previous exposure to *S. stercoralis*, the *S. stercoralis* VirClia^®^ IgG monotest showed high diagnostic accuracy. Its high sensitivity supports current recommendations favouring highly sensitive serological assays for screening and diagnosis in non-endemic clinical settings.

## 1. Introduction

*Strongyloides stercoralis* is a soil-transmitted helminth widely distributed in tropical and subtropical regions, with an estimated 300–600 million infected individuals globally [1,2]. Infection occurs through skin contact with infective larvae (L3) present in soil contaminated with human faeces. Following a tracheobronchial migration phase, parasites reach the small intestines, where adult females reside in the mucosa and reproduce via parthenogenesis, producing eggs that hatch into first-stage larvae (L1), which are eliminated with the feces [3]. Unlike other helminths, *S. stercoralis* is capable of persisting for decades and potentially for life due to its unique auto-infective cycle, whereby a proportion of L1 larvae re-enter the intestinal mucosa or perianal skin [4]. Infection is often asymptomatic or associated with intermittent non-specific symptoms [5]. In immunocompromised hosts, however, the auto-infective cycle may result in an increased larval load, leading to hyperinfection syndrome and disseminated strongyloidiasis. This is characterized by widespread distribution of larvae throughout the body and associated clinical manifestations as well as high mortality [3,4,6]. The life cycle of *S. stercoralis* has important diagnostic implications, as larvae rather than eggs are shed with faeces, often in a limited and intermittent way. As a result, conventional copromicroscopical methods (e.g., direct smears and conventional concentration methods) have low sensitivity [7,8,9,10]. Consequently, a range of alternative diagnostic methods is used, including microscopical techniques based on larval migration (e.g., agar plate culture (APC), Baermann sedimentation), serological assays (e.g., ELISAs targeting antibodies against crude antigens or recombinant *S. stercoralis* antigens), and molecular assays (e.g., real-time PCR for 18S rRNA gene) [11].

The choice of diagnostic strategy depends on the purpose and setting, with different requirements for individual diagnosis in endemic and non-endemic clinical settings versus population-level screening for public health control [11,12,13,14]. In non-endemic clinical settings, screening for strongyloidiasis is recommended for individuals with a risk of previous exposure to *S. stercoralis* (e.g., migrants from endemic areas), as well as for immunosuppressed patients and candidates for immunosuppression [12]. Depending on the patient’s clinical context, recommended diagnostic strategies rely either on serology alone or on a combination of serology and a fecal test for larval detection [12]. Serological methods play an important role because of their high sensitivity [15,16]. Most commercially available serological assays are ELISAs based on crude or recombinant antigens [11,16]. More recently, newer formats have emerged, including the *S. stercoralis* VirClia^®^ IgG monotest (Vircell S.L., Granada, Spain), an automated chemiluminescent immunoassay (CLIA) detecting IgG antibodies against the recombinant NIE and SsIR antigens.

This study aims to evaluate the diagnostic accuracy (sensitivity and specificity) of this novel assay in a non-endemic clinical setting. Given the absence of a gold standard test with perfect sensitivity and specificity for *S. stercoralis,* Bayesian latent class analysis (BLCA) was used.

## 2. Materials and Methods

The Standards for Reporting of Diagnostic accuracy studies using Bayesian latent class models (STARD-BLCM) guidelines were used for reporting methods and results [17] (S1 Info).

### 2.1. Study Design and Sample Set

This monocentric, retrospective study used serum samples from the “Tropica Biobank” (bbmri-eric ID: IT_1605519998080235) of the IRCCS Sacro Cuore Don Calabria hospital (Negrar, Verona, Italy), a referral centre for parasitic diseases in Italy. All samples in the biobank are stored at −80 °C and originate from patients who provided written informed consent to donate for research purposes the leftover volume of the samples collected for clinical purposes.

The following inclusion criteria were applied: (i) serum sample collected from migrants originating from a country endemic for *S. stercoralis* (i.e., countries in Africa, Latin America, South-East Asia, or the Western Pacific region) or collected from patients born in Italy before 1960 with no travel history to a *S. stercoralis* endemic country, (ii) results available for at least one routine serological test for *S. stercoralis* and (iii) if fecal tests were performed, these have been done within 30 days after serum collection. Samples from individuals who received treatment with ivermectin in the previous six months were excluded. To obtain a sample set that mirrors clinical routine work, samples were selected to include individuals mirroring the different combinations of results across the available routine tests observed in our center, showing a broad range of diagnostic profiles, subject to availability of the sample volume required for the test under evaluation. Although the VirClia^®^ monotest itself requires only 10 µL of sample, a larger minimum volume (200 µL) is necessary to enable automated aspiration. Samples were grouped into four geographic populations: (i) Europe, (ii) Africa, (iii) Asia and (iv) America.

### 2.2. The Novel Strongyloides stercoralis VirClia^®^ IgG Monotest

The novel CLIA (Vircell S.L., Granada, Spain) detects IgG antibodies against two recombinant *S. stercoralis* antigens, NIE and SsIR. The assay was performed according to the manufacturer’s instructions using the VirClia^®^ Lotus, a fully automated CLIA analyser allowing non-batched sample processing. The assay is provided in a monodose strip format and includes a calibrator for assay validation. The results are reported qualitatively according to the manufacturer’s predefined cut-off values for the antibody index ((sample antigen relative light units (RLU)—sample blank RLU)/calibrator RLU). The assay was performed blinded to the results of routine diagnostic tests.

### 2.3. Routine Diagnostic Tests for S. stercoralis

Results from previously performed routine diagnostic tests were retrieved from patient records. Routine diagnosis of *S. stercoralis* at the referral centre relies on five tests, selected according to the patient’s clinical context. This includes two serological tests (i.e., an in-house IFAT and a commercial ELISA) and three fecal tests (i.e., classical copromicroscopy after formol-ether concentration, APC and PCR). The in-house IFAT was performed as described by Boscolo et al. (2007) [18]. The commercial ELISA used was the Bordier *Strongyloides ratti* IgG ELISA (Bordier Affinity Products SA, Crissier, Switzerland), which detects IgG antibodies against somatic antigens from larvae of *S. ratti*, and was performed following the manufacturer’s instructions. The procedure for APC followed a modified Koga agar plate method with 10 plates per patient, cultured for 7 days, followed by microscopy performed by an experienced parasitologist [19]. The PCR was a real-time assay performed as described by Verweij et al. (2009) [20].

### 2.4. Sample Size

Target sample sizes were estimated based on the manufacturer’s claimed performance (sensitivity: 94%, specificity: 93%). Using the Wilson score formula with continuity correction, 112 positive cases and 124 negative cases were required to achieve a 95% confidence level with a 10% confidence interval width for sensitivity and specificity, respectively.

### 2.5. Statistical Analyses

Sensitivity and specificity of the novel VirClia^®^ assay were assessed with BLCA, using a fixed-effects type latent class model (S2 Info). Importantly, the five routine diagnostic tests were included for the construction of the model but assessment of their performance estimates was not the aim of the analysis.

Key assumptions of this model are: (1) test properties (sensitivity and specificity) differ between tests but remain constant between individuals and populations for the same test; (2) prevalence differs between populations; and (3) observed test results are independent conditional on the true status of individuals and the correlation structure imposed on the model.

A directed acyclic graph (DAG) was made to support the BLCA (Figure 1) [21]. The DAG visualizes the biological pathways through which exposure to *S. stercoralis* can result in a positive test outcome in a target population of individuals with epidemiological risk of chronic strongyloidiasis. As outlined by Denwood et al. (2024) there are 3 key outcomes of this visualisation process [21]. The first is that the ‘highest common ancestor’ of the DAG approximates the case definition implicitly defined by the choice of tests. The highest common ancestor of the six tests included in this study is ‘exposure to *S. stercoralis* infective larvae’. In the absence of treatment (treatment in the past six months is an exclusion criterion of the study), and assuming successful establishment of infection, exposure can be considered to equal the presence of at least one adult parasitic female worm (Figure 1, red circle). In BLCA, the case definition (Figure 1, red circle) is referred to as the ‘latent class’. The second key outcome of the DAG is the identification of groups of tests with a correlation; independence between direct and indirect assays was assumed, whereas pairwise correlation was allowed between assays within the same group. This resulted in six pairs: PCR-copromicroscopy, PCR-APC and copromicroscopy-APC for the direct methods, and VirClia-IFAT, VirClia-Bordier ELISA and IFAT-Bordier ELISA for the indirect methods. Covariance terms were included for both sensitivity and specificity, giving twelve covariance parameters in total (S2 Info). The third key outcome of the DAG is to identify whether the different biological states, or the strength of the connections between them, is expected to vary between the populations. No such variation was expected between the four geographic populations included.

Informative priors were imposed on the model for the specificity and sensitivity of the Bordier ELISA (Beta (26.94, 13.94) for sensitivity and Beta (10,000, 0.01) for specificity) and the PCR test (Beta (32.02, 25.57) for sensitivity and Beta (441.79, 2.67) for specificity). Note that the prior for the Bordier ELISA specificity is close to deterministic. Prior information was obtained from previously published data on the same tests (ESTRELLA study) (Bordier ELISA: Se: 65.9%, 95%CI 53.4–79.0% and Sp: 100%, 95%CI 99.8–100% & PCR: Se: 55.6%, 95%CI 44.8–67.0% and Sp: 99.4%, 95%CI 98.7–100%)) and calculated using the PriorGen R Package [22,23]. Minimally informative priors (Beta (1,1)) were imposed for all the other test sensitivity and specificity and for the true prevalence parameters.

There were no indeterminate test results, as all completed diagnostic tests yielded strictly binary outcomes (positive or negative). However, missing data were present, as the choice of tests reflected routine clinical practice. No data imputation was conducted; instead, these missing values were natively handled by the Hui-Walter template within the Bayesian framework, which utilizes all available information to estimate parameters without requiring data imputation.

Model adequacy was assessed by comparing observed pairwise test agreement with the agreement predicted by the fitted model (posterior predictive check).

A sensitivity analysis was conducted on the population-level prevalence prior, leaving everything else unchanged (i.e., the informative priors on Bordier ELISA and PCR sensitivity and specificity were not varied). In the primary analysis this prior was minimally informative (Beta (1,1)). Two alternative scenarios were considered, differing only in the assumed infection status of the 26 individuals lacking a fecal test result (Africa 7, America 7, Asia 1, Europe 11), among whom positivity on the VirClia^®^ assay was much higher than in the overall sample (84.6% vs. 49.2%): in scenario B, this positivity is assumed to reflect true infection (prevalence anchors calculated using the Rogan-Gladen estimator); in scenario C, the opposite is assumed, anchoring the prevalence of this subgroup at 0%. For each population, the mean of the alternative prior was calculated as a weighted average between the primary-analysis prevalence and the scenario-specific anchor, weighted by the proportion of the population lacking a fecal test, and re-expressed as a Beta distribution with an equivalent prior sample size of 26. The three scenarios (A: baseline; B; C) were compared in terms of VirClia^®^ sensitivity/specificity and population-level prevalence (median and 95% CrI; details in S6 Info).

All statistical analyses were performed in R (version 4.4.3) using the runjags package (version 2.2.2-5). Bayesian latent class models were run using a Markov chain Monte Carlo method implemented using JAGS 4.3.2 software. Full details of the model are provided in S2 Info.

## 3. Results

### 3.1. Individual Test Results

The mean age of the study population was 41.6 years (SD: 20.4), and 157 (64.1%) patients were male. Routine diagnostic testing was performed according to each patient’s clinical context, and this resulted in varying combinations of available and missing test results. S3 Info provides an overview of the individual result patterns, per geographic population. Cross-tabulation of the tests’ results is reported in S4 Info. The individual-level dataset is available in S7 Info.

A total of 245 samples were included and tested using the novel *S. stercoralis* VirClia^®^ IgG monotest. A test result was obtained for 244 samples (99.59%), while one sample failed automated processing due to insufficient volume for aspiration. Additional diagnostic coverage was as follows: 73.47% of individuals had previously been tested with the Bordier ELISA, 79.59% with the in-house IFAT, 69.39% with copromicroscopy after formol–ether concentration, 71.43% with APC, and 42.86% with PCR.

### 3.2. Estimated Prevalence in the Four Populations

The study population included individuals from four geographical regions: Africa (n = 85), America (n = 31), Asia (n = 53), and Europe (n = 76). Estimated latent class prevalence differed across populations, with median (95% credible interval (CrI)) estimates of 69.2% (58.9–78.7%) in the African population, 75.4% (59.5–89.4%) in the American population, 20.6% (10.1–31.6%) in the Asian population, and 28.3% (18.5–39.2%) in the European population. These prevalence estimates should not be interpreted as representative of the screened populations; rather, they were checked to ensure compliance with the BLCA assumption of a different prevalence across populations.

### 3.3. Diagnostic Accuracy of the Novel VirClia^®^ IgG Monotest

The *S. stercoralis* VirClia^®^ IgG monotest showed a sensitivity of 95.0% (95% CrI: 90.1–98.2%) and a specificity of 90.8% (95% CrI: 85.1–96.0%) in this non-endemic clinical setting. Figure 2 presents the complete diagnostic test result patterns for 80 individuals (32.7% of the study population) testing positive for at least one direct fecal method. Each row corresponds to a unique combination of test outcomes and missing data, with the number of individuals varying across patterns. The novel VirClia^®^ assay yielded positive results for all individuals with at least one positive fecal test result (one sample failed analysis). Diagnostic test result patterns for individuals with either negative results on all performed fecal tests (n = 139) or no available fecal test results (n = 26) are shown in S5 Info.

## 4. Discussion

This study evaluated the diagnostic accuracy of the novel *S. stercoralis* VirClia^®^ IgG monotest in a non-endemic clinical setting. In such settings, screening for strongyloidiasis is recommended for individuals with a risk of previous exposure to *S. stercoralis* (e.g., migrants from endemic areas), as well as for immunosuppressed patients and candidates for immunosuppression [12]. The VirClia^®^ IgG monotest showed a high estimated sensitivity of 95.0% (95% CrI: 90.1–98.2%), indicating that the assay is well suited for identifying infected individuals, minimizing false-negative results. Notably, the high sensitivity was not achieved at the expense of specificity, which was high (90.8% (95% CrI: 85.1–96.0%)). Importantly, the VirClia^®^ assay yielded positive results for all individuals with at least one positive fecal test result. In clinical settings, high sensitivity is particularly important, as missing an infection may have severe consequences because *S. stercoralis* can persist for decades and potentially for life due to its unique auto-infective cycle and may lead to life-threatening hyperinfection syndrome and disseminated strongyloidiasis after immunosuppression [3,4,6]. In addition, current European recommendations for screening infectious diseases among newly arrived migrants advocate serological screening for *S. stercoralis*, based on the rationale that serological assays generally demonstrate higher sensitivity than conventional fecal methods [24]. However, it is a common finding to observe negative results in seroassays in a proportion of individuals who are positive in fecal tests, even when they are immunocompetent [23]. This was not observed for the VirClia^®^ assay, which was positive for all individuals with at least one positive fecal test result (n = 80, one sample failed analysis). In immunocompromised patients, the sensitivity of antibody-based tests may be reduced, and it is therefore recommended to complement negative serological results with fecal diagnostic methods [24]. Although serology is recommended by the European Centre for Disease Prevention and Control as a single diagnostic category, it should be noted that considerable heterogeneity exists among existing serological assays [16]. These differences result from variation in antigen selection (e.g., crude versus recombinant), assay format (e.g., ELISA versus CLIA), the populations and use case for which the assays have been developed, and the imperfect reference tests against which they were evaluated. Furthermore, even though some serological assays may demonstrate high sensitivity, agreement between different serological tests can be poor [23] and therefore, combining a sensitive fecal test with serology may be preferable for screening newly arrived migrants.

In the coming years, it will become increasingly important to move away from considering and recommending serology as a single uniform category. This is particularly relevant also as the WHO has recently developed guidelines for new diagnostic tools for the public health control of strongyloidiasis, highlighting the importance of specificity for population-level surveillance [14,25]. New assays entering the market may therefore be developed for different purposes. While assays intended for individual patient management in clinical settings will prioritize sensitivity to minimize missed infections, assays developed for population-level surveillance will focus on specificity to meet the requirements of the WHO target product profile [25].

Several limitations of this study related to the sample set should be considered. A first limitation is that sample selection was not random or based on a consecutive sampling approach, but rather informed by the availability of previous results, sample availability in the biobank, and adequate sample volume, despite care being taken to mirror the range of clinical statuses observed at the referral centre. However, this selection approach is not expected to bias the estimated diagnostic accuracy of the VirClia^®^ assay since sample selection was independent of its test result. A second limitation is that the patients were screened with different test combinations, reflecting clinical routine work. Fecal testing was missing for 26/245 individuals, among whom VirClia^®^ positivity was markedly higher (84.6% vs. 49.2%), raising the suspicion of selective omission. However, the sensitivity analysis shows that VirClia^®^ sensitivity and specificity remained substantially unchanged across the three scenarios (Se 94.9–95.1%, Sp 90.6–91.0%). While this does not show that the missingness mechanism is benign, it bounds its potential consequence on the accuracy estimates within the scenarios examined. A third limitation is that the study did not include known immunosuppressed patients and candidates for immunosuppression, limiting the generalizability of the results to this population. A last limitation, unrelated to the sample set, is that the estimate of the VirClia^®^ specificity is conditional on a near-perfect specificity of the Bordier ELISA, carried over from previously published data on the same test [23].

A first strength of the study is that the assay was evaluated in the target population and clinical context in which it is intended to be used. This is particularly important as diagnostic sensitivity and specificity may vary substantially depending on the population and setting in which the test is applied. Furthermore, the use of a DAG (Figure 1) provides a clear and transparent definition of the target condition (latent class in the BLCA) and the biology underlying the diagnostic tests used in the analysis. A second strength of this study is the use of BLCA instead of evaluating the assay against conventional methods. Fecal methods are known to have limited sensitivity because larval excretion in stool is often low and intermittent. When a new serological test is evaluated against a fecal test with low sensitivity, which is anyhow treated as the perfect reference standard, truly infected individuals with positive serology but negative stool examination are incorrectly classified as false-positive serological results, leading to an underestimation of specificity of the new test. By modelling the unobserved infection status directly, BLCA overcomes this imperfect reference standard problem and yields less biased estimates of diagnostic accuracy.

## 5. Conclusions

In conclusion, as a fully automated CLIA, the *S. stercoralis* VirClia^®^ IgG monotest offers several practical advantages over manual serological assays, although it requires an initial investment and ongoing maintenance costs. The test demonstrated high diagnostic accuracy in a non-endemic clinical setting. Its high sensitivity makes it a valuable tool for screening individuals at risk of strongyloidiasis in accordance with current recommendations [12,24]. As the assay has only recently become commercially available, further evaluations in different laboratories and patient populations are needed to confirm that its performance remains consistent across settings.

## Figures and Tables

**Figure 1:**
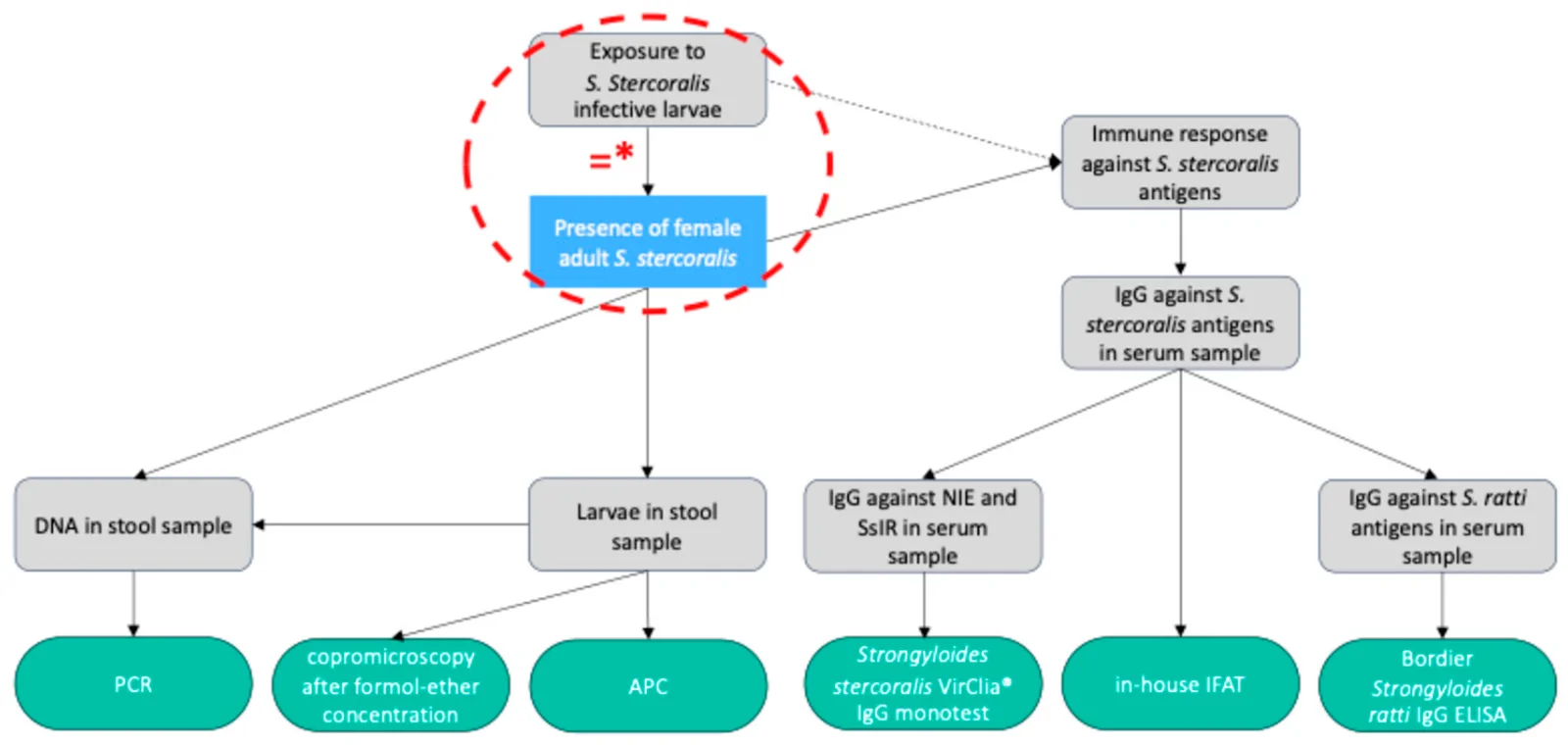
**Directed acyclic graph to support the BLCA.** Biological relationship between the stated target condition (blue), unobserved states (grey), and test results (green) for the six tests used in this study, for one analyzed blood sample and one analyzed stool sample. The approximate case definition (latent class in the BLCA) is circled in red. * Assuming successful establishment of infection following exposure and no ivermectin treatment during the six months preceding diagnosis, exposure can be assumed to equal the presence of at least one adult female worm. Figure based on the design from Denwood et al. (2024) [21]. APC: agar plate culture.

**Figure 2:**
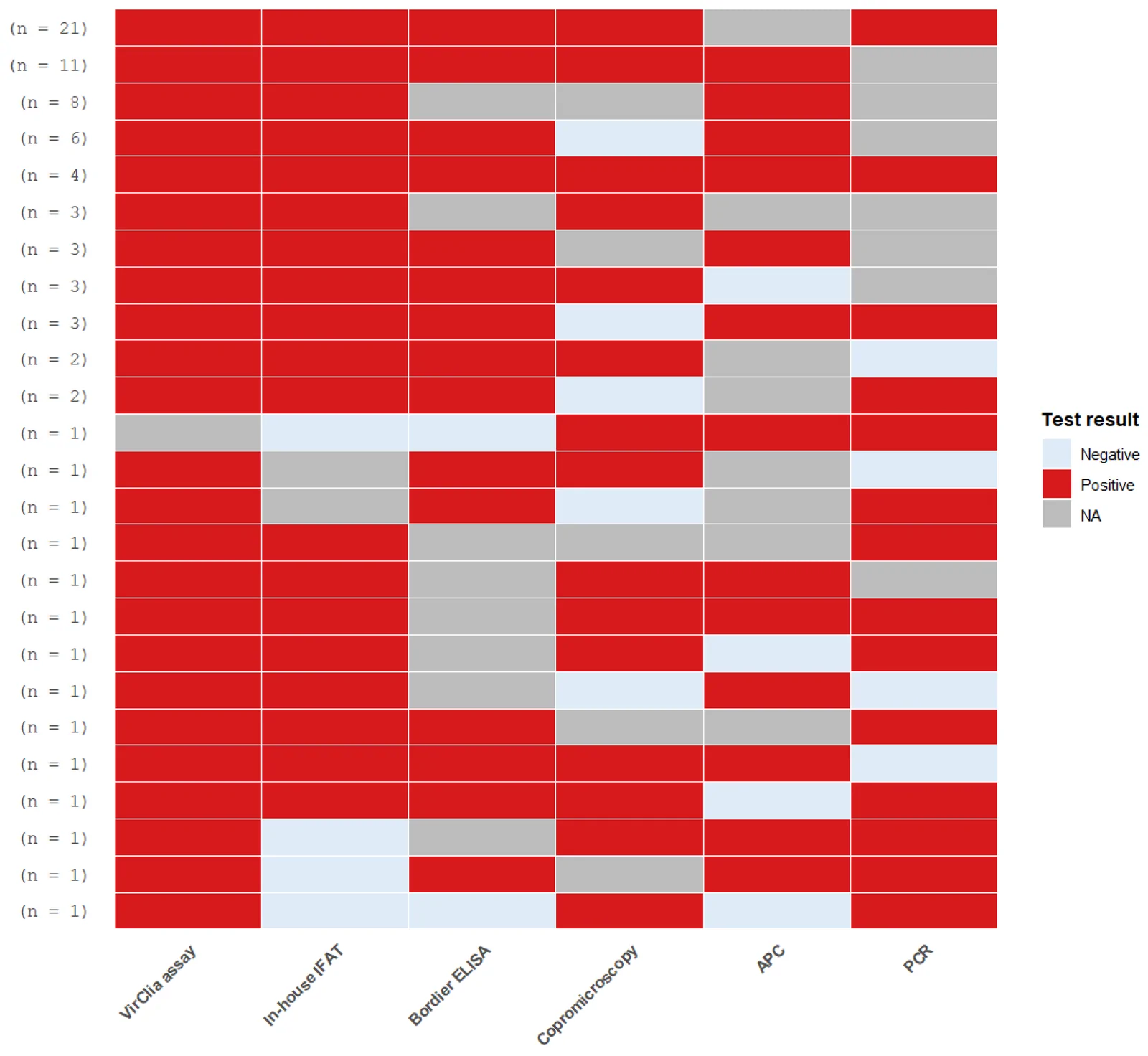
**Pattern of diagnostic test results among individuals testing positive for at least one direct fecal method (n = 80).** Red indicates positive results, blue negative results, and grey missing data. Each row represents a unique combination of test results and missing data. n: number of tested individuals for each combination. The six included tests for *S. stercoralis* are: the VirClia^®^ IgG monotest, an in-house IFAT, the Bordier *S. ratti* IgG ELISA, copromicroscopy after formol-ether concentration, agar plate culture (APC) and PCR.

## Data Availability

All data supporting the findings of this study are included within the article and its Supplementary Information. Further inquiries can be directed to the corresponding author.

## Supplementary Materials

The following supporting information can be downloaded at: https://www.mdpi.com/article/10.3390/diagnostics16193227/s1. **S1 Info**: The Standards for Reporting of Diagnostic accuracy studies using Bayesian latent class models (STARD-BLCM) checklist. **S2 Info**: Full details on the Bayesian latent class analysis performed. **S3 Info**: Pattern of individual diagnostic test results, per geographic population. **S4 Info**: Cross-classified results of the six diagnostic tests for *Strongyloides stercoralis* included in the Bayesian latent class model. **S5 Info**: Pattern of diagnostic test results among individuals with either negative results on all performed fecal tests (n = 139) or no available fecal test results (n = 26). **S6 Info**: Sensitivity analysis of VirClia^®^ IgG monotest sensitivity (Se), specificity (Sp), and population-specific prevalence estimates under three alternative priors for the 26 VirClia^®^-positive samples without fecal testing. **S7 Info**: Individual-level dataset. Anonymized test results. The six included tests for *S. stercoralis* are: the VirClia^®^ IgG monotest, an in-house IFAT, the Bordier *S. ratti* IgG ELISA, copromicroscopy after formol-ether concentration, agar plate culture (APC) and PCR.

## Abbreviations

The following abbreviations are used in this manuscript:

APC	Agar Plate Culture
BLCA	Bayesian Latent Class Analysis
CLIA	Chemiluminescent Immunoassay
CrI	Credible Interval
DAG	Directed Acyclic Graph
ELISA	Enzyme-Linked Immunosorbent Assay
IFAT	Indirect Fluorescent Antibody Test
IgG	Immunoglobulin G
JAGS	Just Another Gibbs Sampler
L1	First-stage Larvae
L3	Third-stage Larvae
PCR	Polymerase Chain Reaction
RLU	Relative Light Unit(s)
rRNA	Ribosomal Ribonucleic Acid
Se	Sensitivity
Sp	Specificity
STARD-BLCM	Standards for Reporting of Diagnostic Accuracy Studies using Bayesian Latent Class Models
WHO	World Health Organization
