# Supplementary material for: Diagnostic Accuracy of a Novel Chemiluminescent Immunoassay for Strongyloides stercoralis in a Non-Endemic Clinical Setting
Source: Diagnostics (Basel). 2026 Oct 6;16(19):3227. doi: 10.3390/diagnostics16193227 (PMC13663916; doi:10.3390/diagnostics16193227)
Supplement: Supplementary file 1 [file diagnostics-16-03227-s001.zip › S1 Info.pdf]

S1 Info - STARD-BLCM checklist

Section & Topic	No	Item	Reported on page #
**TITLE OR ABSTRACT**			
	**1**	Identification as a study of diagnostic accuracy, using at least one measure of accuracy (such as sensitivity, specificity, predictive values, or AUC) **and Bayesian latent class models**	1
**ABSTRACT**			
	**2**	Structured summary of study design, methods, results, and conclusions (for specific guidance, see STARD for Abstracts)	1
**INTRODUCTION**			
	**3**	Scientific and clinical background, including the intended use and clinical role of the **tests under evaluation**	1-2
	**4**	Study objectives and hypotheses, **such as estimation of diagnostic accuracy of the tests for a defined purpose through BLCM**	1-2
**METHODS**			
Study design	**5**	Whether data collection was planned before the **tests** were performed (prospective study) or after (retrospective study)	2
Participants	**6**	Eligibility criteria **and description of the source population**	2-3
	**7**	On what basis potentially eligible participants were identified (such as symptoms, results from previous tests, inclusion in registry)	2-3
	**8**	Where and when potentially eligible participants were identified (setting, location and dates)	2-3
	**9**	Whether participants formed a consecutive, random or convenience series	2-3
Test methods	**10**	**Description of the tests under evaluation**, in sufficient detail to allow replication, **and/or cite references**	3
	**11**	Rationale for choosing the **tests under evaluation in relation to their purpose**	3
	**12**	Definition of and rationale for test positivity cut-offs or result categories of **the tests under evaluation**, distinguishing pre-specified from exploratory	3
	**13**	Whether clinical information was available to the performers or readers of **the tests under evaluation**	3
Analysis	**14a**	**BLCM model** for estimating measures of diagnostic accuracy	4, S2 info
	**14b**	**Definition and rationale of prior information and sensitivity analysis**	4, S2 info
	**15**	How indeterminate results **of the tests under evaluation** were handled	4
	**16**	How missing data **of the tests under evaluation** were handled	4
	**17**	Any analyses of variability in diagnostic accuracy, distinguishing pre-specified from exploratory	NA
	**18**	Intended sample size and how it was determined	3
**RESULTS**			
Participants	**19**	Flow of participants, using a diagram	NA
	**20**	Baseline demographic and clinical characteristics of participants	2-3
	**21**	**Not applicable: the distribution of the targeted conditions is unknown, hence the use of BLCM**	NA
	**22**	Time interval and any clinical interventions between **the tests under evaluation**	2-3
Test results	**23**	Cross tabulation of the **tests' results (or for continuous tests results their distribution by infection stage)**	S4 Info
	**24**	Estimates of diagnostic accuracy **under alternative prior specification** and their precision (such as 95% **credible/probability intervals**)	7, S6 Info
	**25**	Any adverse events from performing **the tests under evaluation**	NA
**DISCUSSION**			
	**26**	Study limitations, including sources of potential bias, statistical uncertainty, and generalisability	7
	**27**	Implications for practice, including the intended use and clinical role of **the tests under evaluation in relevant settings (clinical, research, surveillance etc.)**	1, 2, 7, 8
**OTHER INFORMATION**			
	**28**	Registration number and name of registry	NA
	**29**	Where the full study protocol can be accessed	Fully included in method section
	**30**	Sources of funding and other support; role of funders	Funding section
