# Supplementary material for: Diagnostic Accuracy of a Novel Chemiluminescent Immunoassay for Strongyloides stercoralis in a Non-Endemic Clinical Setting
Source: Diagnostics (Basel). 2026 Oct 6;16(19):3227. doi: 10.3390/diagnostics16193227 (PMC13663916; doi:10.3390/diagnostics16193227)
Supplement: Supplementary file 1 [file diagnostics-16-03227-s001.zip › S2 Info.pdf]

S2 Info - Full details on the Bayesian latent class analysis performed.

1. Model specification

To evaluate the performance of six diagnostic tests for *Strongyloides stercoralis* in the absence of a gold standard, a Bayesian Latent Class Model (BLCM) was fitted using Markov chain Monte Carlo (MCMC) estimation. The model was also used to estimate the infection prevalence across the four populations considered (Europe, Africa, Asia, and America). Following the methodological framework described by Olsen et al. (2022) [2], we applied an adapted Hui-Walter formulation [3] assuming a binary latent state for each case. Given the fact that the range of diagnostic tests used covered different aspects of the infection process including both detection of the parasite and antibody responses to the parasite, we believe that this binary latent state reflects something very close to true *S. stercoralis* infection positive status and true negative status.

To account for potential conditional dependence among the diagnostic tests evaluated in this study, a specific covariance structure was formally defined. Statistical dependence was modeled by explicitly pairing tests that share similar biological pathways, which could consequently lead to correlated errors. Specifically, the model allows for the estimation of covariance parameters for both sensitivity (Active_Se = TRUE) and specificity (Active_Sp = TRUE) across the following test pairs: Vircell and IFAT; Vircell and Bordier ELISA; IFAT and Bordier ELISA; Stool microscopy and Agar plate culture; Stool microscopy and PCR; and Agar plate culture and PCR. Accounting for these covariances relaxes the strict assumption of conditional independence, thereby ensuring more robust and unbiased estimates of the true diagnostic accuracy of the tests.

Informative priors were imposed on the model for the specificity and sensitivity of the Bordier ELISA test (Beta (26.94, 13.94) for sensitivity and Beta (10000,0.01) for specificity) and PCR test (Beta (32.02, 25.57) for sensitivity and Beta (441.79, 2.67) for specificity). This prior information was based on previously published data from the ESTRELLA Study [4] (Bordier ELISA: Se: 65.9%, 95%CI 53.4%-79.0% and Sp: 100%, 95%CI 99.8%-100% & PCR: Se: 55.6%, 95%CI 44.8%-67.0% and Sp: 99.4%, 95%CI 98.7%-100%)) and calculated using the PriorGen R Package [5]. Because the published specificity of the Bordier ELISA was 100%, the resulting prior, Beta (10000,0.01) is close to deterministic; consequently, the posterior estimate reproduces the prior (S2 Info Table 1) and the data are not informative for this parameter. Minimally informative priors (Beta (1,1)) were imposed for all the other test sensitivity and specificity and for the true prevalence parameters. Furthermore, a sensitivity analysis was conducted on the population-level prevalence priors, leaving all other model specifications unchanged (i.e., the informative priors on Bordier ELISA and PCR sensitivity and specificity were not varied).

Statistical analyses were performed in R version 4.4.3. [6]. The model code was generated using the template_huiwalter function from the runjags R package (version 2.2.1-5) [7] and implemented in JAGS [8]. Two Markov Chain Monte Carlo (MCMC) chains with different initial starting values were used to run the model. Inferences were based on 50000 iterations with a thinning interval of 10, after discarding an initial burn-in of 4000 iterations. Thus, a total of 50000 iterations, was used to estimate the posterior distribution. Median posterior and associated 95% Credible Interval (CI) estimates were reported. MCMC chain convergence was assessed using the Gelman-Rubin statistic and trace plots for each parameter. Finally, overall model fit was evaluated using a posterior predictive check, comparing observed pairwise agreement between tests pairs with the agreement predicted by the fitted model across posterior draws (**Fig 1**).

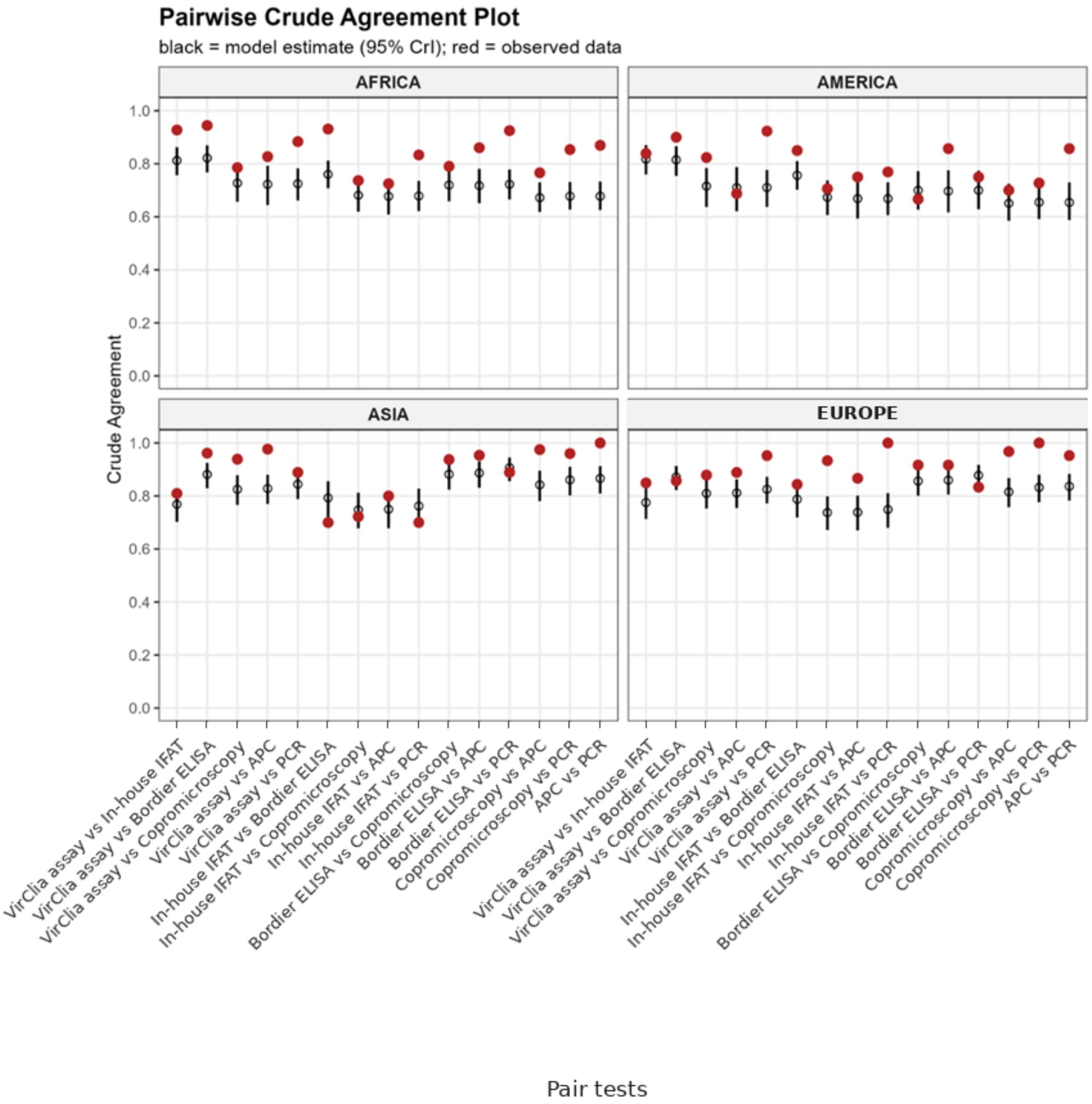

Fig 1. Pairwise crude agreement plot. Model adequacy was assessed by comparing observed pairwise test agreement with the agreement predicted by the fitted model (posterior predictive check).

2. Sensitivity and specificity of the five routine diagnostic tests

The five routine diagnostic tests were included for the construction of the model but assessment of their performance estimates was not the aim of the analysis.

	Sensitivity	Specificity
In-house IFAT	88.2% (81.8–93.9%)	80.8% (71.9–88.8%)
Bordier *Strongyloides ratti* IgG ELISA	81.8% (74.9–88.1%)	99.9% (99.9–100.0%)
PCR	66.6% (58.0–74.8%)	98.7% (97.6–99.5%)
Copromicroscopy after formol-ether concentration	68.4% (58.7–77.8%)	95.4% (90.9–98.7%)
Agar plate culture	67.3% (56.1–78.0%)	96.2% (92.5–98.9%)

Table 1. Posterior medians and 95% credible intervals for sensitivity and specificity of the five routine diagnostic tests. Posterior estimates of all twelve covariance parameters median = 0; 95% CrI within [-0.001, 0.001] for all twelve parameters.

3. References

1. Denwood M, Nielsen SS, Olsen A, Jones HE, Coffeng LE, Landfried G, et al. All that glitters is not gold: An interpretive framework for diagnostic test evaluation using Ascaris lumbricoides as a conceptual example. PLOS Neglected Tropical Diseases. 2024;18(9):e0012481. doi: 10.1371/journal.pntd.0012481.
2. Olsen A, Nielsen HV, Alban L, Houe H, Jensen TB, Denwood M. Determination of an optimal ELISA cut-off for the diagnosis of Toxoplasma gondii infection in pigs using Bayesian latent class modelling of data from multiple diagnostic tests. Preventive Veterinary Medicine. 2022;201:105606. doi: https://doi.org/10.1016/j.prevetmed.2022.105606.
3. Hui SL, Walter SD. Estimating the Error Rates of Diagnostic Tests. Biometrics. 1980;36(1):167-71. doi: 10.2307/2530508.
4. Tamarozzi F, Guevara ÁG, Anselmi M, Vicuña Y, Prandi R, Marquez M, et al. Accuracy, acceptability, and feasibility of diagnostic tests for the screening of Strongyloides stercoralis in the field (ESTRELLA): a cross-sectional study in Ecuador. The Lancet Global Health. 2023;11(5):e740-e8. doi: https://doi.org/10.1016/S2214-109X(23)00108-0.
5. Kostoulas Polychronis. PriorGen: Generates Prior Distributions for Proportions [Internet]. 2018. Available project.from: https://CRAN.R- org/package=PriorGen
6. R Core Team (2024). R: A language and environment for statistical computing. (Version 4.3.3). R Foundation for Statistical Computing, Vienna, Austria
7. Denwood MJ. runjags: An R Package Providing Interface Utilities, Model Templates, Parallel Computing Methods and Additional Distributions for MCMC Models in JAGS. Journal of Statistical Software. 2016;71(9):1 - 25. doi: 10.18637/jss.v071.i09.
8. Plummer M, editor JAGS: A program for analysis of Bayesian graphical models using Gibbs sampling 2003.
