# Supplementary material for: Diagnostic Accuracy of a Novel Chemiluminescent Immunoassay for Strongyloides stercoralis in a Non-Endemic Clinical Setting
Source: Diagnostics (Basel). 2026 Oct 6;16(19):3227. doi: 10.3390/diagnostics16193227 (PMC13663916; doi:10.3390/diagnostics16193227)
Supplement: Supplementary file 1 [file diagnostics-16-03227-s001.zip › S4 Info.pdf]

S4 Info - Cross-classified results of the six diagnostic tests for *Strongyloides stercoralis* included in the Bayesian latent class model.

Samples were grouped into four geographic populations: (i) Europe, (ii) Africa, (iii) Asia and (iv) America.

Tests						Populations				
VirClia assay	In-house IFAT	Bordier ELISA	Copromicroscopy	APC	PCR	Africa	America	Asia	Europe	**Total**
-	NA	-	-	-	-	6		14	1	**21**
+	+	+	+	NA	+	17	3	1		**21**
-	NA	-	-	-	NA	4		13	1	**18**
+	+	+	NA	NA	NA	5	4	1	8	**18**
-	-	NA	-	-	NA	3	1		1	**14**
+	+	+	+	+	NA	7	1	3		**11**
-	-	NA	-	-	-	1	2		6	**9**
-	-	-	-	-	-	2	1	1	4	**8**
+	+	NA	NA	+	NA		1		7	**8**
-	-	-	NA	-	-			1	5	**6**
-	-	NA	NA	-	NA				6	**6**
+	+	+	-	+	NA	5		1		**6**
-	-	-	-	-	NA	1	1	2	1	**5**
-	-	-	NA	-	NA	1			4	**5**
-	+	NA	NA	-	NA	1			3	**4**
+	+	+	+	+	+	3		1		**4**
-	+	-	-	-	-	1		2		**3**
-	NA	-	-	NA	NA	1		2		**3**
+	+	+	-	+	+	2	1			**3**
+	+	+	+	-	NA	2	1			**3**
+	+	+	NA	+	NA		1	1	1	**3**
+	+	NA	+	NA	NA	1	1		1	**3**
+	+	NA	NA	-	NA	1	2			**3**
-	-	-	-	NA	NA			1	1	**2**
-	-	+	-	-	-			1	1	**2**
-	-	NA	-	NA	NA	1		1		**2**
-	-	NA	NA	-	-		1		1	**2**
-	+	-	-	-	NA			1	1	**2**
-	+	-	NA	-	NA				2	**2**
+	-	NA	NA	-	-	1			1	**2**
+	-	NA	NA	-	NA		1		1	**2**
+	+	-	NA	NA	NA	2				**2**

+	+	+	-	-	NA	2				**2**
+	+	+	-	NA	+	1	1			**2**
+	+	+	-	NA	NA	1	1			**2**
+	+	+	+	NA	-	1	1			**2**
+	NA	-	-	-	NA	1			1	**2**
-	-	-	-	NA	-			1		**1**
-	-	-	NA	NA	NA				1	**1**
-	-	+	NA	-	-				1	**1**
-	-	+	NA	NA	NA		1			**1**
-	-	NA	NA	NA	NA				1	**1**
-	+	-	NA	NA	NA		1			**1**
-	+	NA	-	-	NA	1				**1**
-	NA	-	-	NA	-			1		**1**
-	NA	-	NA	-	-			1		**1**
-	NA	-	NA	-	NA	1				**1**
+	-	-	-	-	NA				1	**1**
+	-	-	+	-	+		1			**1**
+	-	-	NA	NA	NA				1	**1**
+	-	+	-	-	-			1		**1**
+	-	+	NA	+	+	1				**1**
+	-	+	NA	NA	NA		1			**1**
+	-	NA	-	-	NA				1	**1**
+	-	NA	+	+	+		1			**1**
+	+	-	-	NA	-			1		**1**
+	+	+	+	-	+	1				**1**
+	+	+	+	+	-	1				**1**
+	+	+	NA	NA	+	1				**1**
+	+	NA	-	-	NA				1	**1**
+	+	NA	-	+	-	1				**1**
+	+	NA	+	-	+				1	**1**
+	+	NA	+	+	+	1				**1**
+	+	NA	+	+	NA				1	**1**
+	+	NA	NA	NA	+		1			**1**
+	NA	-	-	-	-	1				**1**
+	NA	+	-	NA	+	1				**1**
+	NA	+	+	NA	-			1		**1**
NA	-	-	+	+	+	1				**1**
		**Total**				**85**	**31**	**53**	**76**	**245**
