# Supplementary material for: Diagnostic Accuracy of a Novel Chemiluminescent Immunoassay for Strongyloides stercoralis in a Non-Endemic Clinical Setting
Source: Diagnostics (Basel). 2026 Oct 6;16(19):3227. doi: 10.3390/diagnostics16193227 (PMC13663916; doi:10.3390/diagnostics16193227)

S5 Info - Pattern of diagnostic test results among individuals with either negative results on all performed fecal tests (n = 139) or no available fecal test results (n = 26).

Red indicates positive results, blue negative results, and grey missing data. Each row represents a unique combination of test results and missing data. N: number of tested individuals for each combination. The six included tests for *S. stercoralis* are: the VirClia® IgG monotest, an in-house IFAT, the Bordier *S. ratti* IgG ELISA, copromicroscopy after formol-ether concentration, agar plate culture (APC) and PCR.

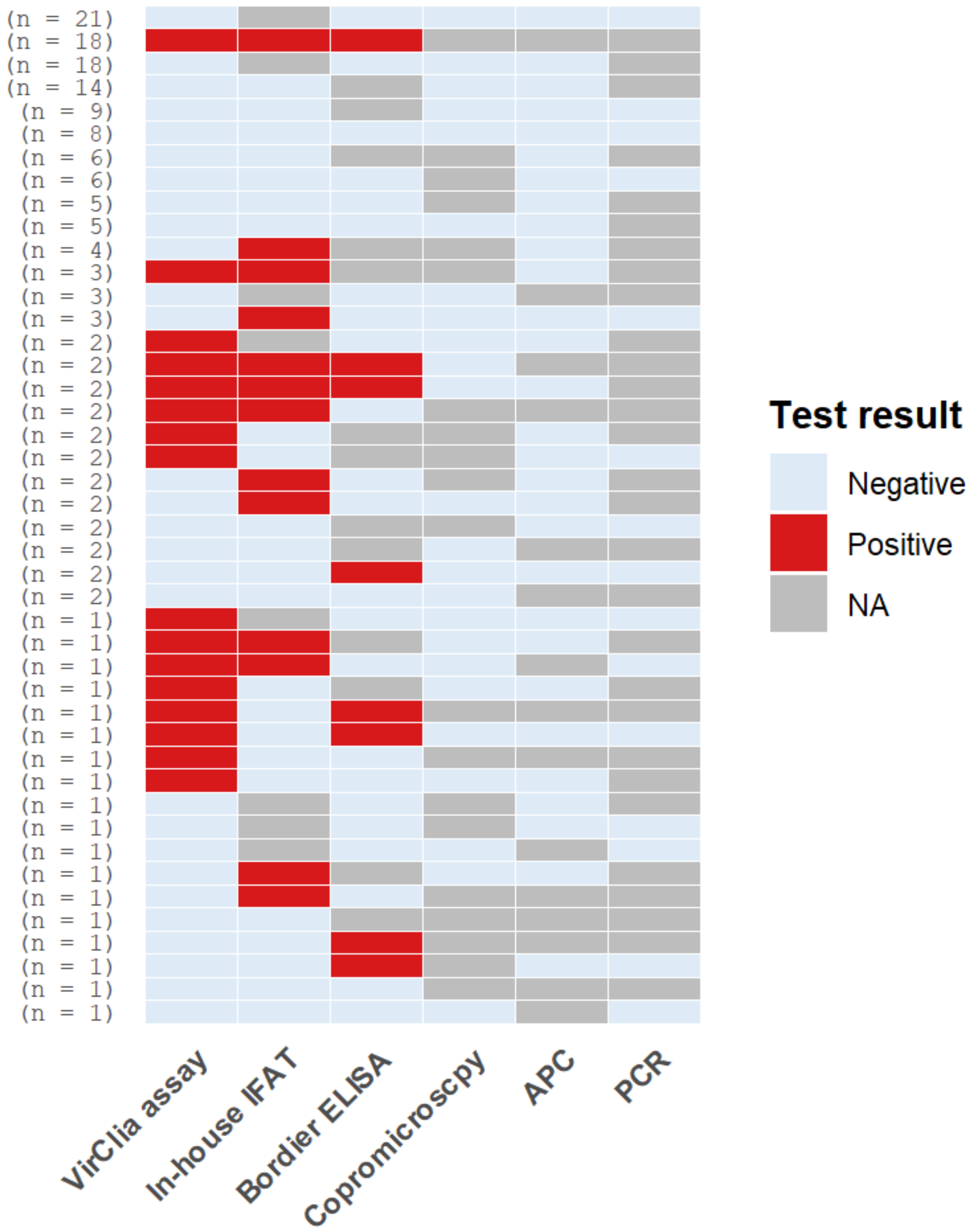

Supplement: Supplementary file 1 [file diagnostics-16-03227-s001.zip › S5 Info.pdf]
