# Supplementary material for: Diagnostic Accuracy of a Novel Chemiluminescent Immunoassay for Strongyloides stercoralis in a Non-Endemic Clinical Setting
Source: Diagnostics (Basel). 2026 Oct 6;16(19):3227. doi: 10.3390/diagnostics16193227 (PMC13663916; doi:10.3390/diagnostics16193227)

S6 Info - Sensitivity analysis of VirClia® IgG monotest sensitivity (Se), specificity (Sp), and population-specific prevalence estimates under three alternative priors for the 26 VirClia®-positive samples without fecal testing.

Points show posterior medians and bars show 95% credible intervals. A: baseline prior, Beta(1,1); B: unconfirmed VirClia®-positive samples assumed true infection; C: unconfirmed VirClia®-positive samples assumed non-infected.

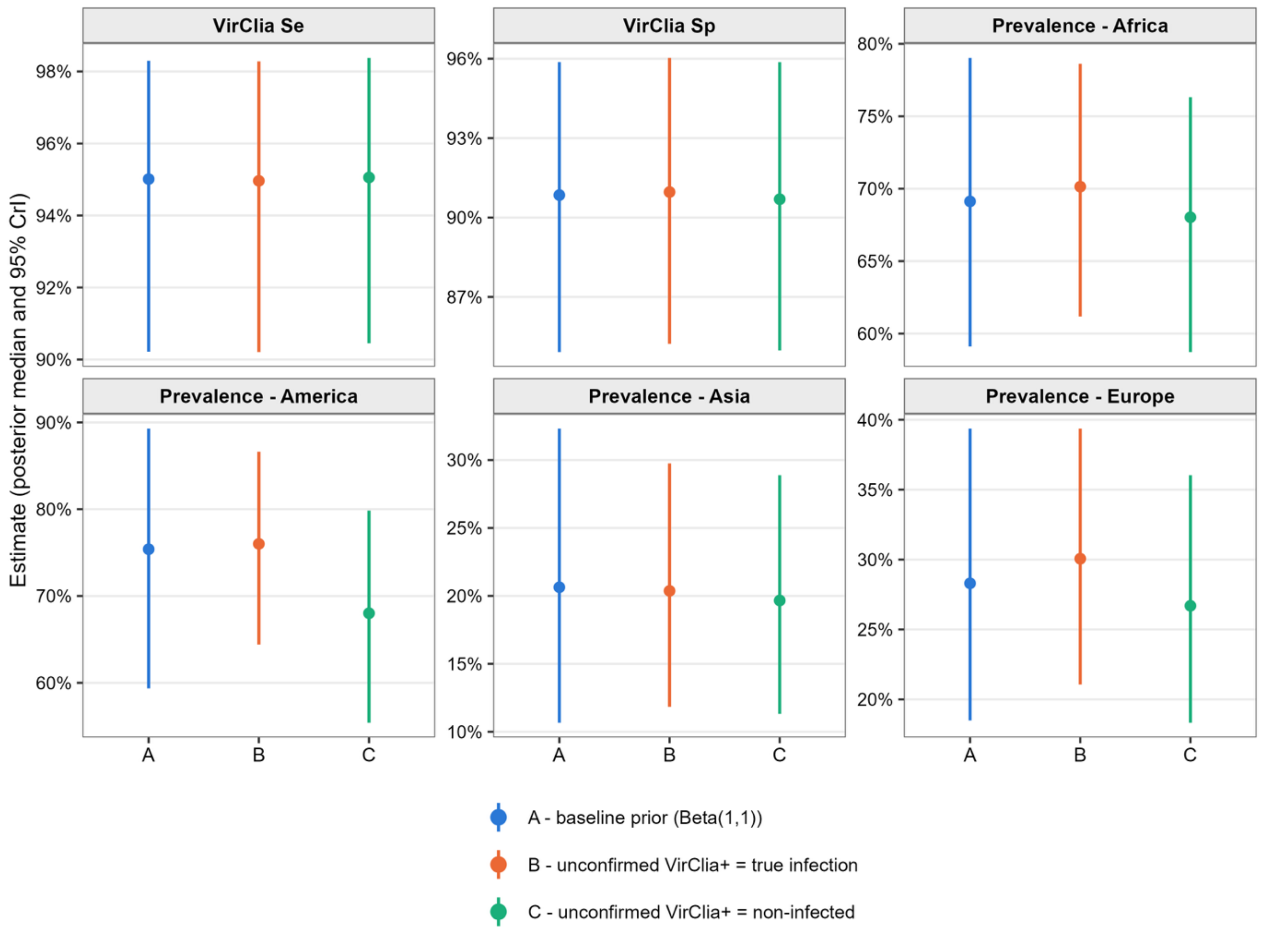

Supplement: Supplementary file 1 [file diagnostics-16-03227-s001.zip › S6 Info.pdf]
